# The Resolutions Adopted

**Published:** 1921-02-05

**Authors:** 


### Policy of King Edward's Hospital Fund.

### THE RESOLUTIONS ADOPTED.

The following are the resolutions adopted:

Resolved, that the President and General Council, without committing themselves on any of the points of comparative detail discussed in the Report of the Executive Committee appended hereto, do hereby adopt the following Statements numbered i to xiii as an outline of the policy of King Edward's Hospital Fund in the present financial difficulties of the hospitals of London:

### General.

i. That the voluntary system of hospital management and control should be preserved as being the most efficient method of providing at the least cost the best medical and surgical treatment combined with advance in medical knowledge and practice.

ii. That a substantial portion at least of the cost of the hospitals should be met by voluntary contributions.

iii. That the present receipts from voluntary contributions are not adequate to meet the present cost of the London voluntary hospitals, to say nothing of the discharge of debts or the provision of necessary extensions.

iv. That any method of increasing income should be such as not to stop voluntary contributions or do away with voluntary management.

v. That any policy or absence of policy which stopped voluntary contributions and did away with voluntary management would bring upon the public funds not only the cost of the hospitals but also additional cost of paid management and of general supervision by some central public department.

### Voluntary Sources of Additional Income.

vi. That a better response to appeals for voluntary contributions is likely when the present uncertainties as to the future of voluntary hospitals are removed.

### Methods of Supplementing Voluntary Income.

vii. That voluntary contributions will need to be supplemented, at all events temporarily, by some other sources of revenue.

viii. That amongst possible methods, as to which experience is being accumulated, and of which the exploration should be encouraged, are included—

(a) various forms of contributions from patients in consideration of treatment received;

(b) various forms of regular contributions from prospective patients as a kind of quasi-insurance or partial patients' payment in advance; and

(c) payment by Government or other public authorities in respect of the treatment of any classes of patients for whom those authorities have taken responsibility.

ix. That, while direct grants from the State in consideration of their general work might endanger voluntary contributions and voluntary management, some form of assistance based on the amount received for the benefit of hospitals from voluntary sources, or some concession by way of abatement of income-tax or death duties, proportioned to gifts, might prove practicable, and might serve to elicit a larger revenue than is now thus obtained.

### Control and Direction of Expenditure.

x. That while it is necessary to increase the income of the hospitals, attention should continue to be paid to the possibility of further economies in expenditure by strengthening the internal financial control in each hospital; by encouraging co-operation among hospitals wherever this is likely to prove advantageous; and by securing that the best possible use is made of all the various forms of voluntary hospital accommodation and equipment, present and future.

### Central Body.

xi. That the necessity for some central organisation, distinct alike from the hospitals and from the public authorities, will be greater in the future even than in the past; that

King Edward's Hospital Fund, the Council of which already includes, amongst others, persons holding representative positions in connection with national and metropolitan government, with the City of London, with religious bodies, and with the medical profession, has in the course of its work as a collecting and distributing agency gradually developed some of the functions of a central administrative body; that it keeps in close touch with the individual hospitals, while retaining its independence; and that it has the confidence of the subscribing public.

xii. That King Edward's Hospital Fund is thus fitted to become a central administrative body for the metropolitan area, with provision for co-operation, in matters of general policy, with a separate central administrative body for extra-metropolitan hospitals; the functions of the King's Fund to include for London the investigation of hospital administration, together with the receipt and distribution of voluntary contributions, and also, if required, of public grants made in accordance with a scheme consistent with the voluntary system.

### Management of Individual Hospitals.

xiii. That, in determining the powers of the central body and the question of the possible representation, on hospital committees, of public and other bodies making payments to hospitals, the principle of the management of the individual hospitals by voluntary committees, themselves possessing wide powers of independence and initiative, should be safeguarded.

### Approximate Revenue and Expenditure Statistics for 1920.

It must be remembered that the accounts of the hospitals for the year 1920 are not yet completed and audited; and that the following figures are only approximations. The Executive Committee of the King's Fund express their great indebtedness to the hospital officers who, by a special effort, have furnished them with the material for an estimate so early in the year.

| | 1920. £ | Corresponding Figures for 1919. £ |
|---|---|---|
| (*a*) The Total Expenditure at 113 London Hospitals for the year 1920 was about ... ... ... ... ... ... ... | 2,841,000 | 2,348,000 |
| (*b*) The Income from Normal Sources, including £153,700 from the King's Fund (being the proportion given to maintenance of hospitals out of the total ordinary distribution of £200,000) and also including in some cases the proceeds of special appeals for the reduction of debt, was about ... | 2,447,000 | 2,117,000 |
| (*c*) In the absence of additional non-recurrent sources of assistance, therefore, the Net Aggregate Deficit for the Year, *i.e.*, the amount still required to cover the year's expenditure at these 113 London Hospitals (taken as a whole) would have been, according to the figures furnished by the hospitals, about ... ... ... ... ... ... ... | 394,000 | 231,000 |

Note.—Largely as the result of the special appeals mentioned in (*b*) above, 42 hospitals had surpluses of income over expenditure during the year. If these are omitted, the deficits for the year at the remaining 71 hospitals with deficits would amount in the aggregate to about £499,000.

| | 1920. £ | Corresponding Figures for 1919. £ |
|---|---|---|
| (*d*) The London Hospitals, however (taken as a whole), also received, to assist them in carrying on during 1920, the Emergency Grants made by the King's Fund on July 5 (out of its accumulated funds, not out of current income received during the year) amounting to ... ... ... ... | 250,000 | |
| (*e*) The net aggregate deficit for the year was thus reduced (owing to this realisation of securities by the King's Fund) to ... ... ... ... ... ... | 144,000 | |
| (*f*) The London Hospitals also (taken as a whole) received grants in December from the National Relief Fund in Reduction of War Deficits incurred during the years 1915 to 1919 inclusive, the amount thus received towards the reduction of past deficiencies of income being ... ... ... ... ... ... ... | 200,000 | |
| (*g*) Even after the receipt of the National Relief Fund grants (besides the proceeds of special appeals already included in income from normal sources in item (*b*) above) there were debts outstanding on December 31 at 59 hospitals, partly as the result of accumulated deficiencies of income and partly as the result of past expenditure on building or improvement. These debts ranged from relatively small sums up to £83,000, and the Total Aggregate Debt of the London Hospitals amounted to about ... ... ... | 544,000 | 681,961 |

(*h*) Besides the amount thus required to meet current expenses and to discharge existing debts the London Hospitals also need a large sum to carry out and maintain various necessary Improvements and Extensions.

